# Vitamin D receptor expression in invasive breast tumors and breast cancer survival

**DOI:** 10.1186/s13058-019-1169-1

**Published:** 2019-07-29

**Authors:** Linnea Huss, Salma Tunå Butt, Signe Borgquist, Karin Elebro, Malte Sandsveden, Ann Rosendahl, Jonas Manjer

**Affiliations:** 1 Department of Surgery, Lund University, Skåne University Hospital, SE-205 02 Malmö, Sweden; 2 Department of Oncology, Aarhus University, Aarhus University Hospital, DE-8000 Aarhus C, Denmark; 3 Division of Oncology and Pathology, Department of Clinical Sciences, Lund University, Skåne University Hospital, SE-221 85 Lund, Sweden

**Keywords:** Vitamin D receptor, Breast cancer, Survival, Mortality, Tissue microarray

## Abstract

**Background:**

Vitamin D has been suggested to prevent and improve the prognosis of several cancers, including breast cancer. We have previously shown a U-shaped association between pre-diagnostic serum levels of vitamin D and risk of breast cancer-related death, with poor survival in patients with the lowest and the highest levels respectively, as compared to the intermediate group. Vitamin D exerts its functions through the vitamin D receptor (VDR), and the aim of the current study was to investigate if the expression of VDR in invasive breast tumors is associated with breast cancer prognosis.

**Methods:**

VDR expression was evaluated in a tissue microarray of 718 invasive breast tumors. Covariation between VDR expression and established prognostic factors for breast cancer was analyzed, as well as associations between VDR expression and breast cancer mortality.

**Results:**

We found that positive VDR expression in the nuclei and cytoplasm of breast cancer cells was associated with favorable tumor characteristics such as smaller size, lower grade, estrogen receptor positivity and progesterone receptor positivity, and lower expression of Ki67. In addition, both intranuclear and cytoplasmic VDR expression were associated with a low risk of breast cancer mortality, hazard ratios 0.56 (95% CI 0.34–0.91) and 0.59 (0.30–1.16) respectively.

**Conclusions:**

This study found that high expression of VDR in invasive breast tumors is associated with favorable prognostic factors and a low risk of breast cancer death. Hence, a high VDR expression is a positive prognostic factor.

**Electronic supplementary material:**

The online version of this article (10.1186/s13058-019-1169-1) contains supplementary material, which is available to authorized users.

## Introduction/background

An enlarging body of research suggests that relatively low levels of vitamin D are associated with a poor breast cancer prognosis [1–4]. In the only study to date using pre-diagnostic levels of vitamin D, we found that women with high vitamin D levels were also at high risk of breast cancer death, compared to women with intermediate levels [5].

Vitamin D exerts its function through the vitamin D receptor (VDR), a nuclear receptor that modulates transcription of target genes, [6] and is to be found in lobule and ductal epithelial cells in normal mammary glands [7, 8]. Compared to normal breast tissue, breast cancer lesions have been found to express more VDR [9]. Since women with intermediate vs low levels of vitamin D may have a better survival following breast cancer, it could be assumed that VDR expression in breast tumors is also associated with a better prognosis.

Only a few studies have reported on breast cancer VDR expression in relation to tumor prognostic factors and breast cancer survival. These studies have shown differing results [10–15], and most of them investigated a rather limited number of breast tumors. The largest study to date showed associations with some tumor-related prognostic factors, but not with survival [10].

In the present study, immunohistochemical staining of VDR was performed on over 700 primary, invasive, breast tumors from the Malmö Diet and Cancer Study (MDCS) [16]. VDR expression was studied in relation to established tumor-related prognostic factors and breast cancer-specific mortality. The hypothesis was that breast cancers with VDR expression would be associated with less aggressive tumors and a low risk of breast cancer death, i.e., a better survival.

## Material and methods

### The Malmö Diet and Cancer Study (MDCS)

The Malmö Diet and Cancer Study is a prospective cohort study which during the time period 1991–1996 included citizens of Malmö, the third largest city in Sweden. All women born between 1923 and 1950 were invited to participate, and 43% of eligible women completed baseline examinations and a questionnaire about socioeconomic factors, previous disease, and medications. Eventually, 17,035 women were included in the cohort and written informed consent was obtained from all participants. The ethical committee in Lund, Sweden, approved the MDCS (LU 51-90) and the present study (Dnr 652/2005 and Dnr 23/2007).

### Study population

**Fig. 1:**
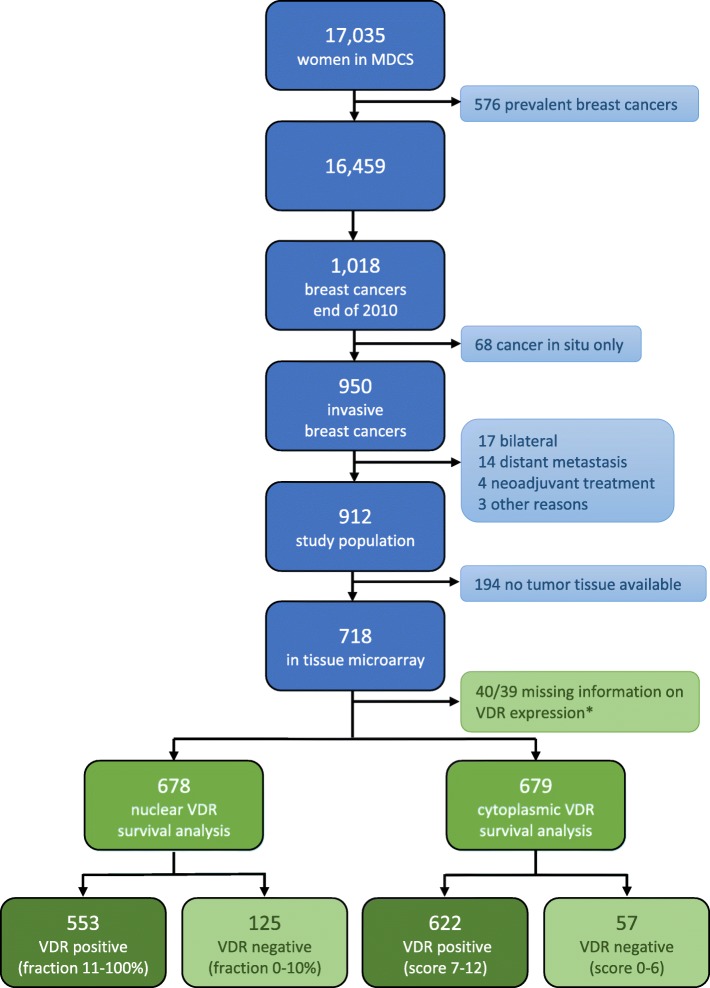
Flowchart of the study population, inclusion and exclusion. Asterisk indicates that it was not possible to score VDR in the nuclei of 40 core pairs and in the cytoplasm in 39 core pairs

Women included in the MDCS were followed using the Swedish Cancer Registry until December 31, 2010, and the Swedish cause of death registry up until December 31, 2016. Since 576 women out of the 17035 had already been diagnosed with breast cancer prior to baseline examination, these women were excluded from the present study. During the follow-up until December 31, 2010, 1018 women were diagnosed with breast cancer. The intention was to investigate tumor characteristics in relation to breast cancer mortality, and due to this, 68 patients with cancer in situ were excluded, since these tumors are associated with a very low breast cancer mortality, if any. Also, patients who had received neoadjuvant treatment (*n* = 4), had distant metastases at diagnosis (*n* = 14), or died from breast cancer-related causes within less than 0.3 years from diagnosis (*n* = 2) were excluded, as well as one woman who declined treatment for 4 years. Bilateral cases (*n* = 17) were also excluded due to difficulties in interpreting tumor characteristics. The final study population consisted of 912 patients (Fig. 1).

### Histopathological analysis and clinical information

Medical records and pathological reports were used to retrieve information on size and laterality of tumors as well as axillary lymph node involvement. Invasive tumors diagnosed during 1991–2004 were pathologically re-evaluated as regards to invasiveness, histological type, grade, estrogen receptor (ER) status, and progesterone receptor (PgR) status by one senior pathologist [17, 18]. Such information was readily available from pathological reports dating 2005 and onwards, with no need for re-evaluation. Information on human epidermal growth factor-2 (HER2) as well as proliferation index (Ki67) on tumors dating 1991–2007 was assessed using tissue microarrays (TMAs) [19]. From 2008 and onwards, information on HER2 and Ki67 status was also retrieved from the diagnostic pathology report. ER and PgR were considered positive at a cut-off of > 10% positively stained nuclei. Results from in situ hybridization (ISH) were used to define HER2 status when available. When immunohistochemistry (IHC) evaluation was used to define HER2 status, HER2 was considered positive when annotated 3+ and negative for 0 or 1+. IHC scores of 2+ were categorized as missing if ISH was not used to confirm the result [16]. For Ki67, the distribution was noted to differ between tumors diagnosed at different periods of time. Therefore, tumors were classified into low, intermediate, or high Ki67 expression based on tertiles within the diagnostic period: 1991–2004, 2005–2007, and from 2008 and onwards.

Based on histological grade; ER, PgR, and HER2 status; and Ki67 category, tumors were classified into molecular subtypes: Luminal A-like, Luminal B-like, HER2 positive, and triple negative, according to criteria used locally within the south Swedish health care region [20]. Luminal A-like tumors were defined as ER positive, HER2 negative, and either (a) histological grade 1, (b) histological grade 2 and low Ki67, or (c) histological grade 2, intermediate Ki67, and positive PgR status. Luminal B-like tumors were also ER positive and HER2 negative but associated with either (a) histological grade 3, (b) histological grade 2 and high Ki67, or (c) histological grade 2, intermediate Ki67, and negative PgR. Regardless of histological grade and hormone receptor status, all HER2-positive tumors were categorized as HER2 positive. All tumors considered ER negative, PgR negative, and HER2 negative were classified as triple negative (ibid.).

Clinical notes were used to retrieve information on the type of breast surgery, surgery to the axillary lymph nodes, and planned adjuvant therapy, as recommended by a multidisciplinary treatment conference following surgery.

### Vitamin D receptor expression

Tumors diagnosed before the end of 2010 were included in the TMAs used for the present study. Out of the 912 patients included in the cohort, tumor tissue was available from 718 tumors. Two 1-mm cores from each tumor were used for the construction of the TMAs (Beecher, WI, USA). Sections of 4 μm were cut and baked on glass slides in a heat chamber for 1 h at 60 °C. Deparaffination and antigen retrieval was performed using PT Link system (Agilent/Dako A/S). The mouse monoclonal D-6 antibody (sc-13133, Santa Cruz Biotechnology) was selected for immunohistochemical analyses of VDR since previous research has found this antibody superior to alternatives as regards to specificity and sensitivity [21, 22]. The antibody was diluted 1:300, and staining was performed automatically in Autostainer *Plus* (Agilent/Dako A/S), with visualization kit K801021-2 (Agilent/Dako A/S) and also counterstained with Mayer’s hematoxylin for 2 min. An automated system was used for taking images of the slides, which were thereafter incorporated in the web-based digital pathological platform PathXL Xplore (http://www.pathxl.com, PathXL Ltd., UK). Microscopic evaluations were performed using PathXL, consistently on the same computer screen.

**Fig. 2:**
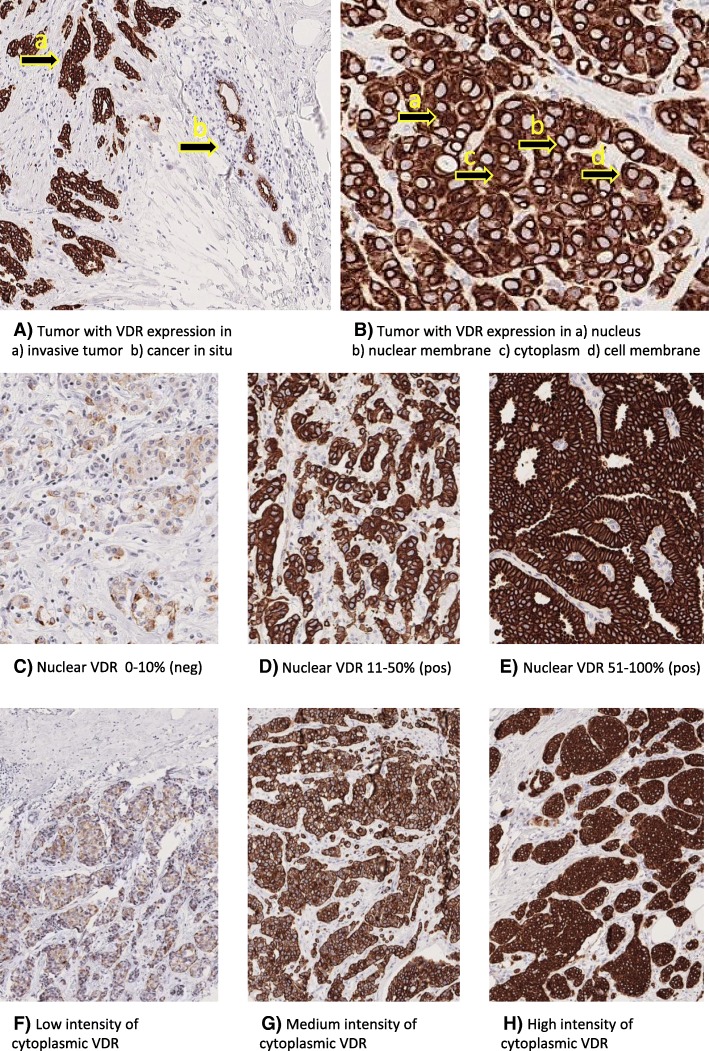
A–H Examples of VDR expression in tissue microarray

After staining, it was noted that VDR was expressed in several subcellular locations of breast cancer cells (Fig. 2A–H). Staining was observed within the nucleus, in the nuclear membrane, in the cytoplasm, and in the cellular membrane. Semi-quantitative scoring was performed evaluating percentages of positive cells for all locations, 0, 1–10%, 11–50%, 51–75%, and 76–100%. Also, the intensity of staining was evaluated as percentages of highly intense stain in nuclei and nuclear membranes. The intensity of cytoplasmic stain was evaluated on four levels: no stain or low, moderate, or high intensity of stain (Fig. 2G, H).

Each core was scored twice by the same observer, at least 4 weeks apart, blinded to clinical and pathological data but with access to information on invasiveness from the previous evaluation of hematoxylin/eosin-stained slides of the cores [16].

Due to considerable differences between the first and second readings of VDR scores regarding nuclear membrane and cellular membrane, it was decided to focus the statistical analysis on the nuclear and cytoplasmic stain. Discordance of nuclear and cytoplasmic stain between core pairs and different readings was treated as described in Appendix 1. The nuclear stain was defined as negative below a cut-off of 10%, positive 11–50%, and strongly positive above 51% of stained nuclei. Cytoplasmic score was calculated by multiplying score for fraction, 0 (0%), 1 (1–10%), 2 (11–50%), 3 (51–75%), and 4 (76–100%), by intensity scores, 0 (no stain), 1 (low intensity), 2 (moderate intensity), and 3 (high intensity). Cytoplasmic scores were thereafter subdivided into three groups, 0–6, 7–9, and 10–11.

### Endpoint retrieval

All women within the MDCS were followed until December 31, 2016, using the Swedish cause of death registry, which provided information on the date of death, cause of death, and underlying and multiple cause of death. When breast cancer was considered the only cause of death or contributing cause of death, the primary endpoint, breast cancer death, was fulfilled. Other women within the cohort were registered as either dead from other causes, emigrated, or alive. End of follow-up was the date of death, date of emigration, or December 31, 2016.

### Statistical analysis

Tumor characteristics and planned treatment were compared between categories of VDR expression. Distributions were compared using the *χ*^2^ test for categorical factors and Kruskal-Wallis test for continuous variables.

Breast cancer mortality (BCM) was calculated as breast cancer-associated death per 100,000 person-years. BCM in relation to VDR expression was tested using a Cox proportional hazards analysis yielding hazard ratios (HRs) with 95% confidence intervals (CI). The assumption of proportional hazards was met as tested by Kaplan-Meier plots, and statistical significance tested by log-rank tests. The crude model was subsequently adjusted in two multivariate models. Model 2 was adjusted for the season of diagnosis and age at diagnosis which is known to affect levels of vitamin D and might therefore influence the VDR expression. Model 3 was further adjusted for tumor factors known to influence breast cancer prognosis, such as the size of tumor, lymph node status, histological type, and molecular subtypes. As the choice of treatment is heavily dependent on prognostic tumor factors included in the adjusted model, it was decided not to adjust also for treatment variables. All adjusted analyses were first performed as complete cases analyses, only including cases with complete information on all included covariates. Missing data on covariates was thereafter included in the adjusted analyses using a multiple imputation model, described in detail in Appendix 2.

Expression of VDR in association with BCM was tested separately for nuclear fraction and cytoplasmic score, both subdivided in three levels of expression and also two levels of expression (negative vs positive). In a sensitivity analysis, negative nuclear fraction was combined with cytoplasmic score 0–6, and this combinatory negative VDR score was tested in a Cox proportional hazards analysis for associations with BCM.

Molecular subtypes showed covariance with VDR expression. To investigate whether or not this covariation explained the association found between VDR expression and BCM, the Cox regression model was repeated stratified by molecular subtypes. Since these groups were small and breast cancer death does not occur in some of the subgroups, the analyses were only performed comparing nuclear VDR negativity vs positivity.

SPSS 25.0 (IBM) was used for all statistical analyses.

## Results

### Patterns of VDR expression

VDR was expressed almost exclusively in tumor cells as compared to surrounding cells within the TMA core. When cancer in situ cells were noted in the same core as invasive tumor cells, in situ cells were differently stained compared to invasive cells. Invasive cancer had a more intense stain and a larger fraction of nuclear staining than cancer in situ cells (Fig. 2A). As only invasive cancer cells were scored for this project, such differences were not systematically recorded.

VDR was expressed in all compartments of cancer cells (Fig. 2B). Nuclear VDR expression was assessable in 678 (94.4%) of tissue core pairs, and cytoplasmic VDR expression as regards to fraction and intensity was scored in 679 (94.6%) of available tumors. Due to difficulties in distinguishing VDR expression between nuclear membrane and cellular membrane, there was a high percentage of discordance between the first and second rounds of scoring at these compartments (14.1% of nuclear membrane fraction and 30.1% of cellular membrane fraction).

Staining patterns of nuclear VDR fraction are illustrated in Fig. 2C–E. Percentages of highly intense stain in nucleus covaried highly with the fraction of nuclear stain and were therefore not included in further analyses. Distribution of nuclear fraction is presented in Table 1.

**Table 1:** Distribution of patient and tumor characteristics in relation to nuclear VDR expression

Eligible cases	All *n* = 912						
Tumor in tissue microarray *n* (%)		Yes 718 (78.7)					No 194 (21.3)
Nuclear VDR assessable *n* (%)		Yes 678 (94.4)				No 40 (5.6)	
Nuclear VDR fraction *n* (%)		Negative 0–10% 125 (18.4)	Positive 11–50% 437 (64.5)	Positive 51–100% 116 (17.1)			
Factor	*n* (%) or mean (SD)	*n* (%) or mean (SD)	*n* (%) or mean (SD)	*n* (%) or mean (SD*)*	*p* value*	*n* (%) or mean (SD)	*n* (%) or mean (SD)
Age at baseline	*56.4 (7.2)*	*55.4 (7.1)*	*56.6 (7.1)*	*55.7 (7.4)*	0.109**	*53.9 (7.5)*	*57.6 (7.0)*
Age at diagnosis	*65.4 (8.1)*	*64.1 (7.7)*	*66.0 (7.8)*	*64.8 (8.6)*	0.026**	*62.1 (9.1)*	*65.9 (8.3)*
Season of diagnosis							
Winter	241 (26.4)	35 (28.0)	116 (26.5)	29 (25.0)	0.646	13 (32.5)	48 (24.7)
Spring	221 (24.2)	38 (30.4)	97 (22.2)	27 (23.3)		9 (22.5)	50 (25.8)
Summer	187 (20.5)	19 (15.2)	96 (22.0)	23 (19.8)		6 (15.0)	43 (22.2)
Fall	263 (28.8)	33 (26.4)	128 (29.3)	37 (31.9)		12 (30.0)	53 (27.3)
BMI at baseline							
< 25	467 (51.2)	60 (48.0)	215 (49.2)	70 (60.3)	0.253	24 (60)	98 (50.5)
≥ 25–30	310 (34)	45 (36.0)	150 (34.3)	33 (28.4)		11 (27.5)	71 (36.6)
≥ 30	135 (14.8)	20 (16.0)	72 (16.5)	13 (11.2)		5 (12.5)	25 (12.9)
Tumor size							
1–10 mm	229 (25.8)	14 (11.2)	93 (21.4)	33 (28.7)	0.002	20 (50)	69 (39.7)
11–20 mm	409 (46.1)	57 (45.6)	217 (49.9)	51 (44.3)		13 (32.5)	71 (40.8)
≥ 21 mm	250 (28.2)	54 (43.2)	125 (28.7)	31 (27.0)		6 (15.0)	34 (19.5)
Unknown	24	0	2	1		1	20
Lymph node status							
Positive	262 (31.9)	50 (41.0)	135 (32.8)	36 (32.4)	0.226	9 (22.5)	32 (22.7)
Negative	559 (68.1)	72 (59.0)	276 (67.2)	75 (67.6)		27 (67.5)	109 (77.3)
Unknown	91	3	26	5		4	53
Nottingham grade							
I	227 (27.2)	7 (5.9)	119 (27.7)	42 (36.5)	< 0.001	12 (30.0)	47 (34.8)
II	393 (47.0)	35 (29.4)	219 (50.9)	61 (53.0)		16 (40.0)	62 (45.9)
III	216 (25.8)	77 (64.7)	92 (21.4)	12 (10.4)		9 (22.5)	26 (19.3)
Unknown	76	6	7	1		3	59
Histological type							
Ductal	596 (70.9)	103 (85.1)	290 (67.8)	84 (72.4)	< 0.001	24 (60.0)	95 (68.3)
Lobular	166 (19.7)	7 (5.8)	113 (26.4)	20 (17.2)		7 (17.5)	19 (13.7)
Other/mixed	79 (9.4)	11 (9.1)	25 (5.8)	12 (10.3)		6 (15.0)	25 (18.0)
Unknown	71	4	9	0		3	55
ER status							
Neg (0–10%)	84 (10.8)	45 (39.5)	25 (6.1)	4 (3.6)	< 0.001	3 (7.5)	7 (6.0)
Pos (> 10%)	694 (89.2)	69 (60.5)	382 (93.9)	106 (96.4)		28 (70.0)	109 (94.0)
Unknown	134	11	30	6		9	78
PgR status							
Neg (0–10%)	311 (41.7)	75 (67.0)	135 (34.5)	39 (36.8)	< 0.001	14 (35.0)	48 (44.9)
Pos (> 10%)	435 (58.3)	37 (33.0)	256 (65.5)	67 (63.2)		16 (40.0)	59 (55.1)
Unknown	166	13	46	10		10	87
HER2							
Neg	646 (90.9)	102 (91.1)	335 (90.3)	91 (91)	0.957	26 (65.0)	92 (92.0)
Pos	65 (9.1)	10 (8.9)	36 (9.7)	9 (9.0)		2 (5.0)	8 (8.0)
Unknown	201	13	66	16		12	94
Ki67							
Low	258 (40.6)	16 (15.8)	150 (45.9)	43 (45.7)	< 0.001	9 (22.5)	40 (44.4)
Intermediate	198 (31.2)	28 (27.7)	104 (31.8)	36 (38.3)		5 (12.5)	25 (27.8)
High	179 (28.2)	57 (56.4)	73 (22.3)	15 (16.0)		9 (22.5)	25 (27.8)
Unknown	277	24	110	22		17	104
Molecular subtypes							
Luminal A-like	350 (55.6)	19 (18.6)	207 (63.1)	62 (66.7)	< 0.001	11 (27.5)	51 (62.2)
Luminal B-like	158 (25.1)	33 (32.4)	76 (23.2)	20 (21.5)		8 (20.0)	21 (25.6)
HER2 positive	65 (10.3)	10 (9.8)	36 (11.0)	9 (9.7)		2 (5.0)	8 (9.8)
Triple negative	56 (8.9)	40 (39.2)	9 (2.7)	2 (2.2)		3 (7.5)	2 (2.4)
Unknown	283	23	109	23		16	112

Percentages do not include missing categories

*p values calculated with *χ*^2^ test if not otherwise noted. All *p* values calculated with only valid categories

**Kruskal-Wallis was used to obtain the *p* value

The vast majority of tumors 624 (91.9%) expressed cytoplasmic VDR to a high fraction (76–100%) of cells. There was a wider distribution of intensity: no stain (*n* = 7, 1.0%), low intensity (*n* = 26, 3.6%), moderate intensity (*n* = 174, 24.2%), and high intensity (*n* = 472, 65.7%) (Fig. 2F–H). Distribution of scores of cytoplasmic VDR expression is presented in Additional file 1: Table S1.

### Covariation of VDR expression and tumor characteristics

Distribution of patient and tumor characteristics in relation to nuclear VDR fraction is presented in Table 1. There was a statistically significant covariation between VDR negativity and many tumor characteristics associated with poor prognosis: large tumor size (*p* = 0.002), high Nottingham grade (*p* < 0.001), negative ER status (*p* < 0.001), negative PgR status (*p* < 0.001), and high Ki67 expression (*p* < 0.001). There was also a statistically significant covariation between histological type and VDR expression, where negative tumors more often were considered ductal (*p* < 0.001). When molecular subtypes were compared, it was noted that only 6.6% of Luminal A-like tumors had a negative VDR expression in the nuclei as compared to 25.6% among Luminal B-like tumors, and 78.4% among triple-negative tumors.

A similar pattern was observed when the distribution of patient and tumor characteristics in relation to cytoplasmic VDR score was analyzed (Additional file 1: Table S1). One difference was that cytoplasmic VDR score also showed statistically significant covariation with HER2, as no tumors within the group of low cytoplasmic score (0–6) were considered HER2 positive (*p* = 0.008).

### Covariation of VDR expression and breast cancer treatment

Mastectomies were performed more often on VDR-negative tumors (55%) compared to VDR-positive tumors (41%). The postoperative treatment conference recommended adjuvant endocrine therapy for a smaller proportion and chemotherapy for a larger proportion of patients with VDR-negative tumors compared to VDR-positive tumors. A similar pattern was seen when cytoplasmic VDR score was compared to treatment factors.

### VDR expression in relation to breast cancer mortality

**Fig. 3:**
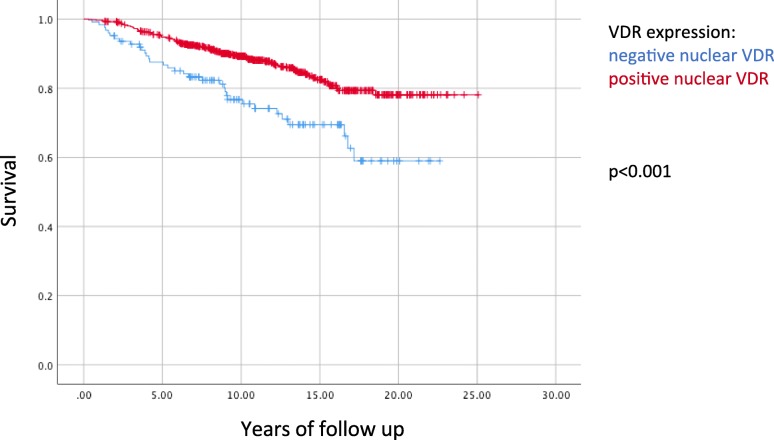
Kaplan-Meier showing breast cancer-specific survival

Mean follow-up was 11.5 years with a standard deviation (SD) of 5.2 years. A Kaplan-Meier analysis confirmed proportional hazards as shown in Fig. 3. Both crude and adjusted analyses showed a statistically significant association between nuclear VDR positivity (a fraction above 10% of stained nuclei) and a low risk of breast cancer-associated death (HR = 0.56, 0.34–0.91) adjusted analysis) (Table 2). The complete case analysis showed similar but not statistically significant results (0.61, 0.35–1.05). Also, similar but not statistically significant results were seen when nuclear VDR fractions 11–50% (0.54, 0.32–0.89) and nuclear VDR fractions 51–100% (0.66, 0.34–1.28) were compared individually to nuclear VDR fraction below 10%. It was also noted that the difference in HR between nuclear VDR fractions 11–50% and nuclear VDR fractions 51–100% was small.

**Table 2:** Vitamin D receptor expression in relation to breast cancer mortality

		Total (*n*)	Person-years	Dead from breast cancer	Breast cancer mortality/100 000	HR^a^	HR^b^	HR^c,#^	HR^c,^*
Nuclear VDR fraction	0–10% (neg)	125	1310	34	2595	1.00 (ref)	1.00 (ref)	1.00 (ref)	1.00 (ref)
	11–100% (pos)	553	6414	77	1200	0.46 (0.31–0.69)	0.42 (0.28–0.63)	0.61 (0.35–1.05)	0.56 (0.34–0.91)
Nuclear VDR fraction	0–10%	125	1310	34	2595	1.00 (ref)	1.00 (ref)	1.00 (ref)	1.00 (ref)
	11–50%	437	5055	61	1206	0.47 (0.31–0.71)	0.41 (0.27–0.63)	0.57 (0.32–1.01)	0.54 (0.32–0.89)
	51–100%	116	1359	16	1177	0.46 (0.25–0.83)	0.44 (0.24–0.79)	0.74 (0.35–1.55)	0.66 (0.34–1.28)
Cytoplasmic VDR score	0–6 (neg)	57	609	17	2791	1.00 (ref)	1.00 (ref)	1.00 (ref)	1.00 (ref)
	7–12 (pos)	622	7137	94	1317	0.47 (0.28–0.79)	0.44 (0.26–0.74)	0.88 (0.39–1.97)	0.59 (0.30–1.16)
Cytoplasmic VDR score	0–6	57	609	17	2791	1.00 (ref)	1.00 (ref)	1.00 (ref)	1.00 (ref)
	7–9	152	1712	25	1460	0.52 (0.28–0.96)	0.50 (0.27–0.92)	1.01 (0.43–2.40)	0.58 (0.27–1.24)
	10–12	470	5425	69	1272	0.45 (0.27–0.77)	0.42 (0.25–0.72)	0.81 (0.36–1.86)	0.59 (0.30–1.18)

^a^Crude analysis

^b^Adjusted for age at and season of diagnosis

^c^Adjusted for same factors as ^b^ but also for size of tumor, lymph node status, histological type, and molecular subtypes

^#^Complete case analysis: analysis including only cases with complete information on all covariates

*Multiple imputation performed to include individuals with missing data on covariates in analysis

As regards to HRs calculated for different cytoplasmic VDR scores, they showed similar results as for nuclear VDR expression, i.e., more VDR expression was associated with decreased risk of breast cancer death, but not statistically significant when adjusted (0.59, 0.30–1.16) (Table 2).

### VDR expression in relation to breast cancer mortality stratified by molecular subtypes

HRs for breast cancer death calculated in groups stratified by molecular subtypes are presented in Table 3. There was a statistically significant association between VDR expression and BCM within the Luminal B-like tumors as nuclear VDR positivity was associated with a decreased risk of breast cancer death (0.37, 0.18–0.77). Also, there seemed to be a possible association within the Luminal A-like molecular subtype and reduced risk of breast cancer death, but this association did not reach statistical significance (0.76, 0.32–2.53). For HER2-positive and triple-negative molecular subtypes, no statistically significant results were observed.

**Table 3:** Vitamin D receptor expression in relation to breast cancer mortality stratified by surrogate molecular subtypes

Surrogate molecular subtype	Nuclear VDR fraction	*n*	Person-years	Dead from breast cancer	Breast cancer mortality/100 000	HR^a^	HR^a,^*	HR^b^	HR^b,^*	HR^c^	HR^c,^*
Luminal A-like	0–10%	19	275	4	1455	1.00 (ref)	1.00 (ref)	1.00 (ref)	1.00 (ref)	1.00 (ref)	1.00 (ref)
	11–100%	269	3132	23	734	0.56 (0.19–1.62)	0.76 (0.27–2.14)	0.44 (0.15–1.31)	0.61 (0.21–1.76)	0.79 (0.24–2.63)^d^	0.76 (0.32–2.53)^d^
Luminal B-like	0–10%	33	325	11	3381	1.00 (ref)	1.00 (ref)	1.00 (ref)	1.00 (ref)	1.00 (ref)	1.00 (ref)
	11–100%	96	1125	15	1334	0.38 (0.18–0.84)	0.37 (0.18–0.76)	0.42 (0.19–0.93)	0.36 (0.17–0.73)	0.43 (0.19–0.94)^e^	0.37 (0.18–0.77)^e^
HER 2 positive	0–10%	10	82	3	3639	1.00 (ref)	1.00 (ref)	1.00 (ref)	1.00 (ref)	1.00 (ref)	1.00 (ref)
	11–100%	45	436	12	2751	0.78 (0.22–2.79)	0.82 (0.25–2.64)	0.74 (0.20–2.74)	0.73 (0.22–2.47)	1.13 (0.31–4.09)^d^	1.00 (0.30–3.33)^d^
Triple negative	0–10%	40	419	10	2388	1.00 (ref)	1.00 (ref)	1.00 (ref)	1.00 (ref)	1.00 (ref)	1.00 (ref)
	11–100%	11	123	3	2440	1.04 (0.28–3.77)	0.85 (0.25–2.86)	0.80 (0.21–3.00)	0.70 (0.21–2.37)	0.87 (0.23–3.19)^e^	0.78 (0.23–2.61)^e^

^a^Crude analysis

^b^Adjusted for age at and season of diagnosis

^c^Hazard ratio adjusted for covariate most affecting the estimate: ^d^lymph node status and ^e^season of diagnosis

*Multiple imputation performed to include individuals with missing data on molecular subtype

## Discussion

In the present study, VDR expression was found to be associated with favorable prognostic characteristics, such as small size, low grade, ER positivity, PgR positivity, low Ki67 expression, and Luminal-like molecular subtypes. This corresponds to the finding that VDR-positive tumors were found to be associated with a decreased risk of breast cancer-specific mortality, but this association was also independent of other prognostic factors.

### Immunohistochemistry and patterns of VDR expression

In the present material, VDR was found almost exclusively in tumor cells. As TMA was targeted to evaluate invasive tumors, there was a scarcity of normal breast cells and thorough evaluation of this matter could not be performed. Previous research have found expression of VDR to be higher in in situ and infiltrative carcinoma compared to benign breast disease or normal tissue [23, 24], but others argue the opposite [25].

When this study was initiated, it was expected to find only nuclear staining concerning VDR, since this is what most previous studies have observed [8, 10, 26]. It was therefore surprising to find VDR staining also in the nuclear membrane, the cytoplasm, and the cellular membrane in our TMA. Early research which concluded VDR to be a primary nuclear receptor used radioactive 1,25-(OH)2 vitamin D_3_ to identify the receptor in mammary cells [8]. More recently, it has been shown that cytoplasmic unliganded VDR present in tumor cells of cell lines and mouse models promotes cell growth in contrast to the inhibitory effects of intranuclear VDR which has been activated by vitamin D [27]. Another recent study also demonstrated VDR to be localized in the cytoplasm of dividing cells [28]. Hence, unliganded VDR can be found on other locations in the tumor cells, and at least one other study on breast cancer survival has also found VDR in the cytoplasm along with the nuclei [12].

When research on which antibody to use in the study, the Santa-Cruz D-6 antibody was preferred over alternatives, since validation of this particular antibody for immunohistochemistry was considered superior to alternatives [21, 22]. This antibody was not used in the previous studies, which is a possible explanation for different staining patterns.

Since VDR was found on multiple subcellular locations in cancer cells, and there were no previous records in the literature about this, we concluded it best to evaluate the fraction of stained cancer cells at all sites. In an attempt also to score the intensity of nuclear and nuclear membrane VDR expression, an additional score of the highly intensely stained fraction was added. This method was based on previous reports that concluded better reproducibility on very strongly positive scores than including intermediate intensities [29]. Considering intensity in the cytoplasm, it was easier to distinguish intensities of intermediate staining pattern at this location, why it was also included. When evaluating results from scoring, we concluded that scores as regards to nuclear fraction and intensity and cytoplasmic fraction and intensity were congruent enough to be valid.

Scores concerning membranous expression had a low reproducibility and were therefore not included in the statistical analyses. It would have been interesting to compare staining in different subcellular compartments in associations to tumor prognostic factors and breast cancer mortality, since VDR activated by vitamin D is translocated into the nucleus and has been shown to reduce the viability of triple-negative breast cancer cell lines [30], inhibit breast cancer cell line growth [31], and induce autophagy in breast cancer cell lines and in normal breast tissue of mice [32]. Another previous study has shown that unliganded VDR in the cytoplasm promotes cell growth in contrast to the inhibitory intranuclear ligand-dependent actions of VDR [27]. We suggest that VDR located in the cellular membrane, not activated by vitamin D, hypothetically may be associated with prognostic factors and BCM differently compared to intranuclear VDR. In our study, there were only few individuals with negative nuclear VDR expression and positive cytoplasmic stain, and therefore, we consider that there was not enough power to find any association between this small group of individuals and a possibly elevated risk of BCM. However, tumors with nuclear VDR expression do probably also express VDR in the cytoplasm.

### Methodological considerations

All Swedish residents are given a unique civil registration number at birth or immigration. It is therefore possible to link all women in our cohort to different registries. The Swedish Cause of Death Registry which was used to retrieve information on the cause of death is reported to be virtually complete on the event of death and to 96% complete to cause of death [33]. Deaths caused by a tumor have been found to be correctly registered in 90% of cases [34].

Analyses were performed with nuclear fraction and cytoplasmic score analyzed separately (Table 3). As regards to cytoplasmic score, there were few individuals with negative scores (*n* = 57), and results from statistical analyses were harder to interpret, although they seemed to be congruent with results from analyzing nuclear VDR fraction. In a sensitivity analysis, a combinatory score of nuclear and cytoplasmic expression was calculated and used to determine HR of BCM. There were very few individuals with both a negative nuclear fraction and negative cytoplasmic score (*n* = 54), and when this group was expanded to contain either a higher cytoplasmic score (7–10) or larger nuclear fraction (11–50%), any effects of VDR negativity could not be observed. Hence, we conclude that in our material a dichotomized variable of a nuclear fraction of VDR expression with a cut-off of 10% is appropriate to use and will be used in our future studies. Another previous study has also noted that the intensity of VDR expression seems to be of less importance [12].

A tissue microarray (TMA) is not an evaluation of a complete tumor, and therefore, results from an immunohistochemical analysis of TMA are not comparable to a diagnostic immunohistochemical analysis. For research purposes, a TMA is valuable as it makes it possible to evaluate many tumors under a comparably short duration of time. Another weakness of a TMA study is that very small tumors are not represented in the TMA, as seen in Table 1, where small tumors accounted for almost 40% of tumors not included in TMA but only approximately 20% of tumors evaluated as regards to nuclear VDR were small.

### VDR and associations with prognostic factors and breast cancer treatment

Tumor factors previously known to predict breast cancer prognosis included in this study was tumor size, lymph node status, tumor grade, histological type, ER and PgR status, HER2 amplification, Ki67 expression, and molecular subtypes derived from the factors above. Covariation of statistical significance was seen with most of the prognostic factors, except with lymph node involvement and HER2 amplification. A recent study, which used computer-assisted image analysis for evaluations of nuclear VDR expression, showed very similar results as regards to covariance with tumor prognostic factors [10], which strengthens our results. Earlier, smaller studies have reported divergent results. One study showed associations with tumor size and lymph node involvement, but not with grading, estrogen receptors, progesterone receptors, or HER2 [12]; others reported no associations [13, 14]. The earlier studies were quite small and did not evaluate many of the associations that we found.

As VDR expression covaried with prognostic factors, it was expected to find associations also with the suggested breast cancer treatment. It was noted that VDR-negative tumors more often were surgically treated with mastectomy (they were larger), less often suggested endocrine therapy, and more often chemotherapy (they were ER negative to a larger extent).

### VDR and breast cancer mortality (BCM)

The present study showed a statistically significant association between BCM and VDR tumor expression among breast cancer patients (HR 0.56, 0.34–0.91) (Table 2), which has not been reported previously. Previous studies have not used BCM as endpoint when evaluating breast cancer prognosis [10–14], and associations found between VDR expression and different endpoints are not conclusive. The most recent and largest study, by Al-Azhri et al., reported very similar results to ours as regards to covariation with tumor prognostic factors, but showed no association between VDR expression and overall survival, progression-free survival, or breast cancer-specific survival [10]. Differences in study population such as a shorter follow-up time (mean 72 months, compared to 137 months in our study) and differing tumor characteristics and that they might have included women with metastatic disease in their study population (not mentioned) may explain some of these discrepancies. The earlier and smaller studies have used different approaches on retrieving information on VDR expression and have not associated VDR expression with any differences in survival [11, 13, 14] although Berger et al. noted that VDR-positive tumors were associated with a longer disease-free interval [11]. Ditsch et al. showed that VDR expression was associated with a between better progression-free survival, and overall survival in univariate analyses [12], which strengthens our results.

Another study which suggested that phenotype of the normal breast tissue surrounding a breast cancer can predict outcome showed that when VDR was expressed along with androgen receptor (AR) and ER in the surrounding breast tissue, patient outcomes were more favorable than when none of those three was expressed [35]. Their results are also in line with ours, suggesting that VDR expression is associated with a better breast cancer prognosis.

Molecular subtypes were included as a covariate in the adjusted model of BCM, which maintained statistical significance when positive nuclear expression (11–100%) was compared to negative nuclear expression (0–10%), although the confidence interval was widened (0.56, 0.34–0.91) (Table 2). The model stratified on molecular subtypes showed that for tumors classified as Luminal B, VDR positivity was associated with a decreased BCM. Since breast cancer deaths were uncommon for women with tumors classified as Luminal A, the results had a poor precision and wide confidence intervals. Also, small numbers of HER2-positive tumors and triple-negative tumors made the analyses regarding VDR expression and breast cancer mortality inconclusive. Still, we believe that the stratified model confirmed that the positive prognostic effect seen with positive VDR expression was not all due to covariation with molecular subtypes.

## Conclusion

The present study indicates that high VDR expression in breast cancer cell nuclei is associated with favorable prognostic factors and a decreased risk of breast cancer death. Women with VDR-positive breast tumors have a better breast cancer-specific survival compared to women with VDR-negative tumors. Future studies ought to investigate the combined effect of VDR expression and serum levels of vitamin D in relation to breast cancer prognosis.

### Additional file

Additional file 1: Distribution of patient and tumor characteristics in relation to cytoplasmic VDR expression. (DOCX 24 kb)

## Data Availability

The data that support the findings of this study are available on request from the corresponding author (LH). The data are not publicly available due to Swedish restrictions.

## Appendix 1

### Description of handling intraindividual scoring differences

Each tumor was represented by two cores in the TMA, and in order to obtain validity of values of expression, scoring was performed twice. At least several weeks, up to months, went between scoring of the same tumor, depending on other obligations of the evaluator (LH). Due to many (seven) scoring categories, an intraindividual discrepancy between the first and second scoring rounds can be expected. Such a discrepancy was treated as follows:

If there were different scores recorded from one core pair in the first evaluation and only one in the second (for example 1,2 and 2,2), the score which was consistent between core pair was recorded. Also, if the score between one core pair differed two steps in one evaluation and the second evaluation showed a score in between (for example 1,3 and 2,2), the second result was recorded. This was considered appropriate since we decided that if there was a discrepancy between cores, the two cores should be evaluated as one entity. When there were no differences between core pairs but (only) one score unit between the first and second evaluation, the second score was considered correct, due to the learning curve during the first evaluation. After this procedure, there were still differences as regards to scores for nuclear fraction (*n* = 30), cytoplasmic fraction (*n* = 18), and cytoplasmic intensity (*n* = 21). These tumors were re-evaluated, with the pair of cores of the same tumor considered as one entity. After this re-evaluation, score for only four tumors could not be concluded, and a second evaluator (AR) was consulted before the score was determined.

Measurement error due to scoring discrepancies was evaluated by performing sensitivity analyses in which all tumors with any discrepancy (either between cores or between first and second evaluation) between negative and positive nuclear VDR expression were excluded (*n* = 62). These analyses were performed without multiple imputation, and results differed only to a minor extent compared to when the differing cases were included. The adjusted analyses differed the most: 0.65 (0.36–1.15) compared to 0.61 (0.35–1.05).

## Appendix 2

### Description of the imputation model

Among 718 tumors included in the TMA, there were missing data on the following variables: tumor size (*n* = 4), lymph node status (*n* = 38), tumor grade (*n* = 17), histological type (*n* = 16), ER status (*n* = 56), PgR status (*n* = 79), HER2 expression (*n* = 107), Ki-67 expression (*n* = 173), and molecular subtypes (*n* = 171). Also, it was not possible to evaluate expression of VDR in the cytoplasm for 39 of tumors and in nuclei for 40 of tumors. All above variables were included in the multiple imputation model, except molecular subtypes which were calculated based on results from imputation. Since the analysis in mind for the multiple imputation model was a Cox regression, baseline hazards and breast cancer death were also included in the model, as well as age and season of diagnosis (covariates in the analysis). Further predictors of imputed values and of missingness, such as BMI and year of diagnosis, were also added. SPSS 25.0 (IBM) was used for multiple imputation, and 30 imputations were made. SPSS uses logistic regression for imputation of categorical variables, and linear regression was used for imputation of baseline hazards. Convergence was checked, and it appeared after two iterations, although ten iterations were used to impute values.

In the Cox analysis only, imputed values for covariates (tumor size, lymph node status, histological type, and molecular subtypes) were used. Only tumors with values based on defined scores of VDR expression were included in the final analyses.

## Abbreviations

BCM: Breast cancer mortality
CI: Confidence interval
ER: Estrogen receptor
HER2: Human epidermal growth factor-2
HR: Hazard ratio
ISH: In situ hybridization
Ki67: Proliferation index factor Ki67
MDCS: Malmö Diet and Cancer Study
PgR: Progesterone receptor
SD: Standard deviation
TMA: Tissue microarray
VDR: Vitamin D receptor
