# Supplementary material for: Vitamin D receptor expression in invasive breast tumors and breast cancer survival
Source: Breast Cancer Res. 2019 Jul 29;21:84. doi: 10.1186/s13058-019-1169-1 (PMC6664551; doi:10.1186/s13058-019-1169-1)
Supplement: Supplementary file 1 — Distribution of patient and tumor characteristics in relation to cytoplasmic VDR expression. (DOCX 24 kb) [file 13058_2019_1169_MOESM1_ESM.docx]

| Supplementary table 1: Distribution of patient and tumor characteristics in relation to cytoplasmic VDR expression | | | | | | | |
| --- | --- | --- | --- | --- | --- | --- | --- |
| Eligible cases | All n=912 |  | | | | |  |
| Tumor in tissue microarray n (%) |  | Yes  718 (78.7) | | | | | No  194 (21.3) |
| Cytoplasmic VDR assessable n (%) |  | Yes  679 (94.6) | | | | No  39 (5.4) |  |
| Cytoplasmic VDR score  n (%) |  | 0-6  57 (8.4) | 7-9  152 (22.4) | 10-12  470 (69.2) |  |  |  |
| Factor | n (%) or  *mean (SD)* | n (%) or  *mean (SD)* | n (%) or  *mean (SD)* | n (%) or  *mean (SD)* | p-value* | n (%) or  *mean (SD)* | n (%) or  *mean (SD)* |
| Age at baseline | *56.4 (7.2)* | *55.5 (6.3)* | *56.3 (7.9)* | *56.3 (7.0)* | 0.777** | *53.8 (7.5)* | *57.6 (7.0)* |
| Age at diagnosis | *65.4 (8.1)* | *65 (7.6)* | *64.9 (8.6)* | *65.7 (7.9)* | 0.565** | *62.2 (8.8)* | *65.9 (8.3)* |
| Season of diagnosis |  |  |  |  |  |  |  |
| Winter | 241 (26.4) | 16 (28.1) | 44 (28.9) | 119 (25.3) | 0.760 | 14 (35.9) | 48 (24.7) |
| Spring | 221 (24.2) | 17 (29.8) | 32 (21.1) | 113 (24.0) |  | 9 (23.1) | 50 (25.8) |
| Summer | 187(20.5) | 10 (17.5) | 27 (17.8) | 101 (21.5) |  | 6 (15.4) | 43 (22.2) |
| Fall | 263 (28.8) | 14 (24.6) | 49 (32.2) | 137 (29.1) |  | 10 (25.6) | 53 (27.3) |
| BMI at baseline |  |  |  |  |  |  |  |
| <25 | 467 (51.2) | 25 (43.9) | 74 (48.7) | 247 (52.6) | 0.490 | 23 (59.0) | 98 (50.5) |
| ≥25-30 | 310 (34.0) | 24 (42.1) | 50 (32.9) | 154 (32.8) |  | 11 (28.2) | 71 (36.6) |
| ≥30 | 135 (14.8) | 8 (14.0) | 28 (18.4) | 69 (14.7) |  | 5 (12.8) | 25 (12.9) |
| Tumor size |  |  |  |  |  |  |  |
| 1-10 mm | 229 (25.8) | 9 (15.8) | 20 (13.2) | 112 (23.9) | 0.020 | 19 (50.0) | 69 (39.7) |
| 11-20 mm | 409 (46.1) | 24 (42.1) | 79 (52.3) | 222 (47.4) |  | 13 (34.2) | 71 (40.8) |
| ≥21 mm | 250 (28.2) | 24 (42.1) | 52 (34.4) | 134 (28.6) |  | 6 (15.8) | 34 (19.5) |
| Unknown | 24 | 0 | 1 | 2 |  | 1 | 20 |
| Lymph node status |  |  |  |  |  |  |  |
| Positive | 262 (31.9) | 19 (33.9) | 52 (35.6) | 150 (33.9) | 0.932 | 9 (25.0) | 32 (22.7) |
| Negative | 559 (68.1) | 37 (66.1) | 94 (64.4) | 292 (66.1) |  | 27 (75.0) | 109 (77.3) |
| Unknown | 91 | 1 | 6 | 28 |  | 3 | 53 |
| Nottingham grade |  |  |  |  |  |  |  |
| I | 227 (27.2) | 2 (3.8) | 30 (20.0) | 135 (29.2) | <0.001 | 13 (36.1) | 47 (34.8) |
| II | 393 (47.0) | 10 (18.9) | 73 (48.7) | 234 (50.6) |  | 14 (38.9) | 62 (45.9) |
| III | 216 (25.8) | 41 (77.4) | 47 (31.3) | 93 (20.1) |  | 9 (25) | 26 (19.3) |
| Unknown | 76 | 4 | 2 | 8 |  | 3 | 59 |
| Histological type |  |  |  |  |  |  |  |
| Ductal | 596 (70.9) | 48 (87.3) | 112 (74.7) | 318 (69) | 0.012 | 23 (60.5) | 95 (68.3) |
| Lobular | 166 (19.7) | 2 (3.6) | 29 (19.3) | 110 (23.9) |  | 6 (16.7) | 19 (13.7) |
| Other/mixed | 79 (9.4) | 5 (9.1) | 9 (6.0) | 33 (7.2) |  | 7 (19.4) | 25 (18.0) |
| Unknown | 71 | 2 | 2 | 9 |  | 3 | 55 |
| ER status |  |  |  |  |  |  |  |
| neg (0-10%) | 84 (10.8) | 30 (58.8) | 20 (14.0) | 24 (5.5) | <0.001 | 3 (10.0) | 7 (6.0) |
| pos (>10%) | 694 (89.2) | 21 (41.2) | 123 (86.0) | 414 (94.5) |  | 27 (90.0) | 109 (94.0) |
| Unknown | 134 | 6 | 9 | 32 |  | 9 | 78 |
| PgR status |  |  |  |  |  |  |  |
| neg (0-10%) | 311 (41.7) | 37 (72.5) | 65 (48.5) | 146 (34.4) | <0.001 | 15 (51.7) | 48 (44.9) |
| pos (>10%) | 435 (58.3) | 14 (27.5) | 69 (51.5) | 279 (65.6) |  | 14 (48.3) | 59 (55.1) |
| Unknown | 166 | 6 | 18 | 45 |  | 10 | 87 |
| HER2 |  |  |  |  |  |  |  |
| neg | 646 (90.9) | 52 (100) | 110 (85.3) | 367 (91.1) | 0.008 | 25 (92.6) | 92 (92.0) |
| pos | 65 (9.1) | 0 (0.0) | 19 (14.7) | 36 (8.9) |  | 2 (7.4) | 8 (8.0) |
| Unknown | 201 | 5 | 23 | 67 |  | 12 | 94 |
| Ki67 |  |  |  |  |  |  |  |
| Low | 258 (40.6) | 4 (8.7) | 38 (33.3) | 167 (46.0) | <0.001 | 9 (40.9) | 40 (44.4) |
| Intermediate | 198 (31.2) | 12 (26.1) | 30 (26.3) | 126 (34.7) |  | 5 (22.7) | 25 (27.8) |
| High | 179 (28.2) | 30 (65.2) | 46 (40.4) | 70 (19.3) |  | 8 (36.4) | 25 (27.8) |
| Unknown | 277 | 11 | 38 | 107 |  | 17 | 104 |
| Molecular subtypes |  |  |  |  |  |  |  |
| Luminal A-like | 350 (55.6) | 4 (8.9) | 54 (46.2) | 229 (63.4) | <0.001 | 12 (50.0) | 51 (62.2) |
| Luminal B-like | 158 (25.1) | 12 (26.7) | 35 (29.9) | 83 (23.0) |  | 7 (29.2) | 21 (25.6) |
| HER2 positive | 65 (10.3) | 0 (0.0) | 19 (16.2) | 36 (10.0) |  | 2 (6.9) | 8 (9.8) |
| Triple negative | 56 (8.9) | 29 (64.4) | 9 (7.7) | 13 (3.6) |  | 3 (13.6) | 2 (2.4) |
| Unknown | 283 | 12 | 35 | 109 |  | 15 | 112 |
| Percentages does not include missing categories.  *P-values calculated with Chi2-test if not otherwise noted. All p-values calculated with only valid categories.  **Kruskal-Wallis used to obtain p-value. | | | | | | | |
